# Holistic Variability Analysis in Resistive Switching Memories Using a Two-Dimensional Variability Coefficient

**DOI:** 10.1021/acsami.2c22617

**Published:** 2023-04-07

**Authors:** Christian Acal, David Maldonado, Ana M. Aguilera, Kaichen Zhu, Mario Lanza, Juan Bautista Roldán

**Affiliations:** † Departamento de Estadística e Investigación Operativa e Instituto de Matemáticas (IMAG), Universidad de Granada, Facultad de Ciencias, Avd. Fuentenueva s/n, 18071 Granada, Spain; ‡ Departamento de Electrónica y Tecnología de Computadores, Universidad de Granada, Facultad de Ciencias, Avd. Fuentenueva s/n, 18071 Granada, Spain; § Physical Science and Engineering Division, King Abdullah University of Science and Technology (KAUST), Thuwal 23955-6900, Saudi Arabia; ∥ Department of Electronic and Biomedical Engineering, Universitat de Barcelona, Martí i Franquès 1, E-08028 Barcelona, Spain

**Keywords:** resistive memories, variability, variability coefficient, functional data analysis, holistic methodology

## Abstract

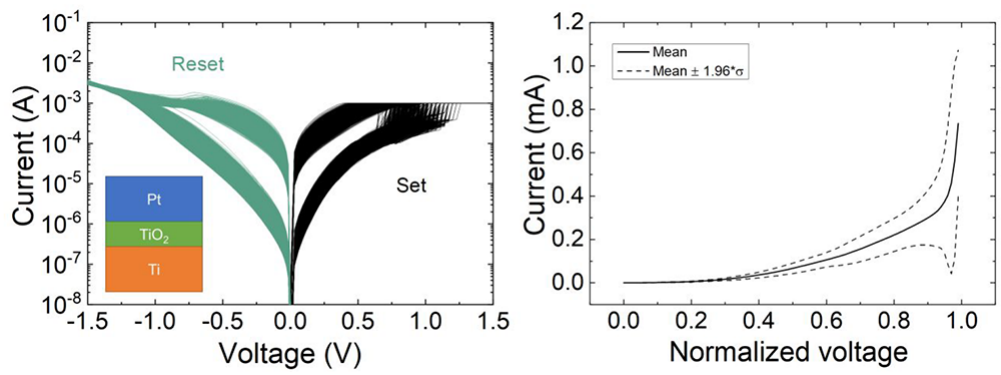

We present a new methodology to quantify the variability of resistive switching memories. Instead of statistically analyzing few data points extracted from current versus voltage (*I*–*V*) plots, such as switching voltages or state resistances, we take into account the whole *I*–*V* curve measured in each RS cycle. This means going from a one-dimensional data set to a two-dimensional data set, in which every point of each *I*–*V* curve measured is included in the variability calculation. We introduce a new coefficient (named two-dimensional variability coefficient, 2DVC) that reveals additional variability information to which traditional one-dimensional analytical methods (such as the coefficient of variation) are blind. This novel approach provides a holistic variability metric for a better understanding of the functioning of resistive switching memories.

## 1 Introduction

Memristors,^1−3^ i.e., resistors whose value can be programmed by applying electrical stresses, are one of the most studied electron devices nowadays because of their excellent electrical performance and easy fabrication, which make them attractive for a great variety of applications in the nanoelectronics landscape. Among these applications, we find nonvolatile memories^3−5^ (TSMC^6^ and INTEL^7^ incorporate these devices in the 22 nm node), neuromorphic computing,^8−17^ and hardware cryptography.^18−21^ Memristors can be fabricated using different types of materials, including metal oxides for resistive memories,^22^ phase-change materials,^23^ magnetic materials,^24^ and ferroelectric materials.^25^

The operation of memristive nonvolatile memories made of metal oxide materials, often termed resistive random access memories (RRAM), is characterized by resistive switching (RS) where charge conduction is linked to internal ion movement and concurrent redox reactions in the dielectric and dielectric/electrode interfaces, which can lead to different resistive states in both a digital and an analog context.^26−28^ Academic studies have shown devices with a good endurance above >10^10^ cycles^29^—although there is no commercial RRAM with an endurance higher than 10^7^ cycles—as well as long data retention time above 10 years and low write energy down to ∼0.1 pJ.^30^ Moreover, their technology is complementary metal–oxide–semiconductor (CMOS) compatible, and the devices can be built in compact crossbar structures (with 4*F*^2^ footprint, where *F* is the minimum technology haft-pitch).^4^

Although the resistive memories are being incorporated at the industrial level, certain issues such as variability have to be addressed to enable their mass production in high-integration-density circuits and/or microchips.^3,31^ In addition to the conventional device-to-device variability, the inherently stochastic^31−34^ operation of RRAMs produces cycle-to-cycle variability.^35^ The characterization of cycle-to-cycle variability is normally evaluated by measuring and statistically analyzing different RS parameters extracted from current versus voltage (*I*–*V*) plots, such as the set and reset voltages (*V*_set_, *V*_reset_), the corresponding set and reset currents (*I*_set_, *I*_reset_), and/or the state resistances (*R*_LRS_: LRS for low resistance state; *R*_HRS_: HRS for high resistance state). The statistical analyses normally consist of cumulative distribution functions (CDFs), coefficients of variation (CV) (calculated as the standard deviation to mean ratio, σ/μ), and the fitting of different distribution functions to the sample to assess the structure of the data.^32,33,36,37^ So far, studies in this field have only employed one-dimensional distributions; e.g., in a 1000 cycles long RS series, the *V*_set_ and *V*_reset_ are obtained, and the CDFs and the CVs of the data set are calculated. In this type of study each *I*–*V* plot is represented by just a single point that corresponds to *V*_set_ or *V*_reset_; however, such an approach has two important problems: (i) some information could be misleading, as two *I*–*V* curves with different shape may have similar *V*_set_ and *V*_reset_—according to the conventional one-dimensional statistical analysis such a situation would not show cycle-to-cycle variability—and (ii) much information, such as changes in curvature trends, is missing.

To avoid this problem, here we show a new two-dimensional holistic methodology to quantify the cycle-to-cycle variability of memristive devices, in which the whole set of *I*–*V* curves are considered. In this manner, different curves with the same *V*_set_ and *V*_reset_ lead to a non-negligible variability. We perform this analysis, which is fully described at the mathematical level in the Supporting Information, using functional data analysis.^37−40^ This study can be considered two-dimensional because each element is a complete *I*–*V* curve instead of a single *V*_set_ and *V*_reset_ value. Consequently, by considering the variability generated all along the *I*–*V* curves, a holistic perspective is achieved, and a more exhaustive variability estimation can be performed.

It is important to highlight that when resistive switching devices are employed in the nonvolatile memory context, they are usually operated by pulsed voltage signals. In this approach the set and reset voltages are key, and this fact could justify a variability analysis focused on these parameters, the current practice at present. However, other applications such as neuromorphic computing require an analogue operation of resistive switching devices.^9−13^ Different conductance levels are used to mimic synaptic plasticity in hardware neural networks. Moreover, in the particular case of spiking neural networks, a type of network that mimics more closely the operation of the biological neural tissue,^41^ the resistive switching device operation is purely analogue. In this latter context, an analysis just based on set and reset voltages does not work to evaluate the appropriateness of a device in terms of variability. Nevertheless, for these types of applications with an analogue operation approach for the devices, the new statistical procedure we propose here, accounting for the whole *I*–*V* curves, provides an optimum variability analysis.

## 2 Experimental Section

**Figure 1:**
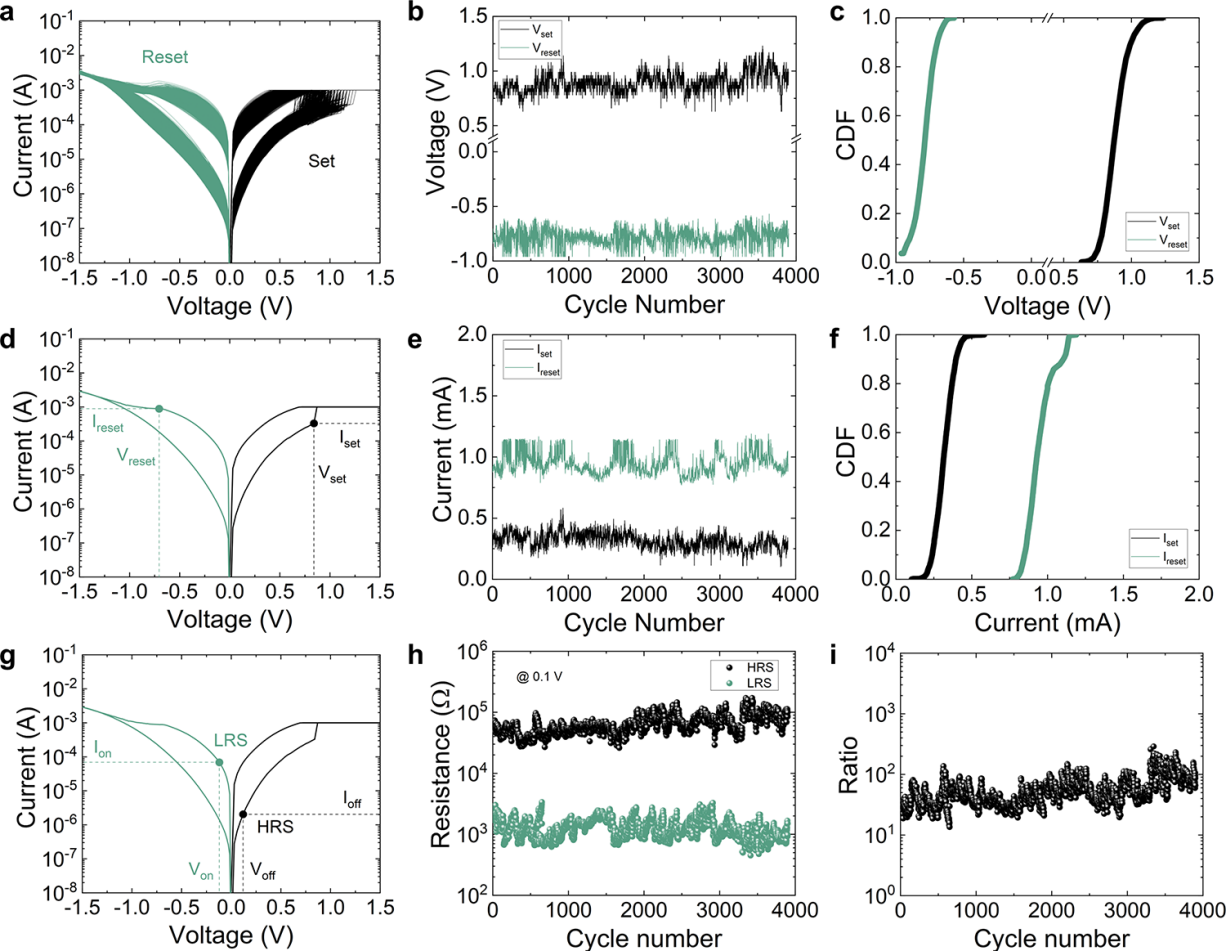
(a) Experimental current versus voltage for the 3900 cycles measured applying a ramped voltage stress in the device based on the Pt/TiO_2_/Ti stack. (b) Experimental values of *V*_set_ and *V*_reset_ versus cycle number for the long RS series. (c) Cumulative distribution functions for the set and reset voltages. (d) Experimental current versus voltage curve for a single cycle detailing the set and reset voltages and currents points. (e) Experimental values of *I*_set_ and *I*_reset_ versus cycle number for the long RS series. (f) Cumulative distribution functions for the set and reset currents. (g) Experimental current versus voltage curve for a single cycle depicting the HRS and LRS points where the resistance is obtained. (h) LRS and HRS resistance versus cycle number for all the measured resistive switching series. The data are read at 0.1 V. (i) HRS/LRS resistance ratio versus cycle number calculated from the data corresponding to (h).

The first group of devices employed for this statistical study is based on a Pt/TiO_2_/Ti stack. The anodic oxidation was conducted potentiostatically by applying a constant voltage (the anodization voltage) to the Ti substrate, which grew an anodic TiO_2_ oxide with thickness of 10 nm. The top electrodes were patterned using a laser-patterned shadow mask and an electron beam evaporator, and the size of the devices studied was 50 μm × 50 μm. More details of the fabrication process are given in ref (42). We measured 3900 consecutive cycles of set and reset processes, as shown in Figure 1a. In these curves, we were able to extract *V*_set_ and *V*_reset_ (Figure 1b) by finding the maximum separation from an imaginary straight line that joins the first point of the *I*–*V* curve measured at *V* = 0 V and the first point where the compliance current for the set process is achieved (*V*_set_) and by localizing the minimum value of the current derivative evaluated at the interval comprised between −0.3 and −1 V (*V*_reset_),^43^ as shown in Figure 1d. The corresponding currents for these voltages versus the RS cycle are shown in Figure 1e. These are one-dimensional data sets whose CDFs can be plotted, as shown in Figures 1c and 1f; CDFs are the conventional plots performed to assess variability in memristive devices. The coefficients of variation were calculated for the set and reset voltages distributions (assuming absolute values) (see Table 1).

**Table 1:** Single-Point Functional Coefficient of Variation and Conventional Coefficient of Variation for the Two Technologies under Study

variability metric	Pt/TiO_2_/Ti stack	Au/Ti/TiO_2_/Au stack
2DVC_f_(set)	0.178	0.2036
2DVC_r_(set)	0.0523	0.0422
2DVC_t_(set)	0.0506	0.0425
CV(set)	0.0980	0.0878
2DVC_f_(reset)	0.0399	0.2371
2DVC_r_(reset)	0.0260	0.3532
2DVC_t_(reset)	0.0254	0.2219
CV(reset)	0.0979	0.2055

The figure of merit that evaluates the switching ratio performance is also calculated (see panels g–i). We highlight these results, in particular the on–off ratio (*R*_HRS_/*R*_LRS_) for the Pt/TiO_2_/Ti device. The data are read at 0.1 V (see Figure 1g). The resistance levels exhibit great stability, in both the LRS and HRS, along the resistive switching series. The on–off ratio is shown in Figure 1i. The value is nearly constant (this means a good cycle-to-cycle variability for this ratio) to 10^2^ for most of the resistive switching series. Therefore, the technology is suitable for nonvolatile memory applications.

**Figure 2:**
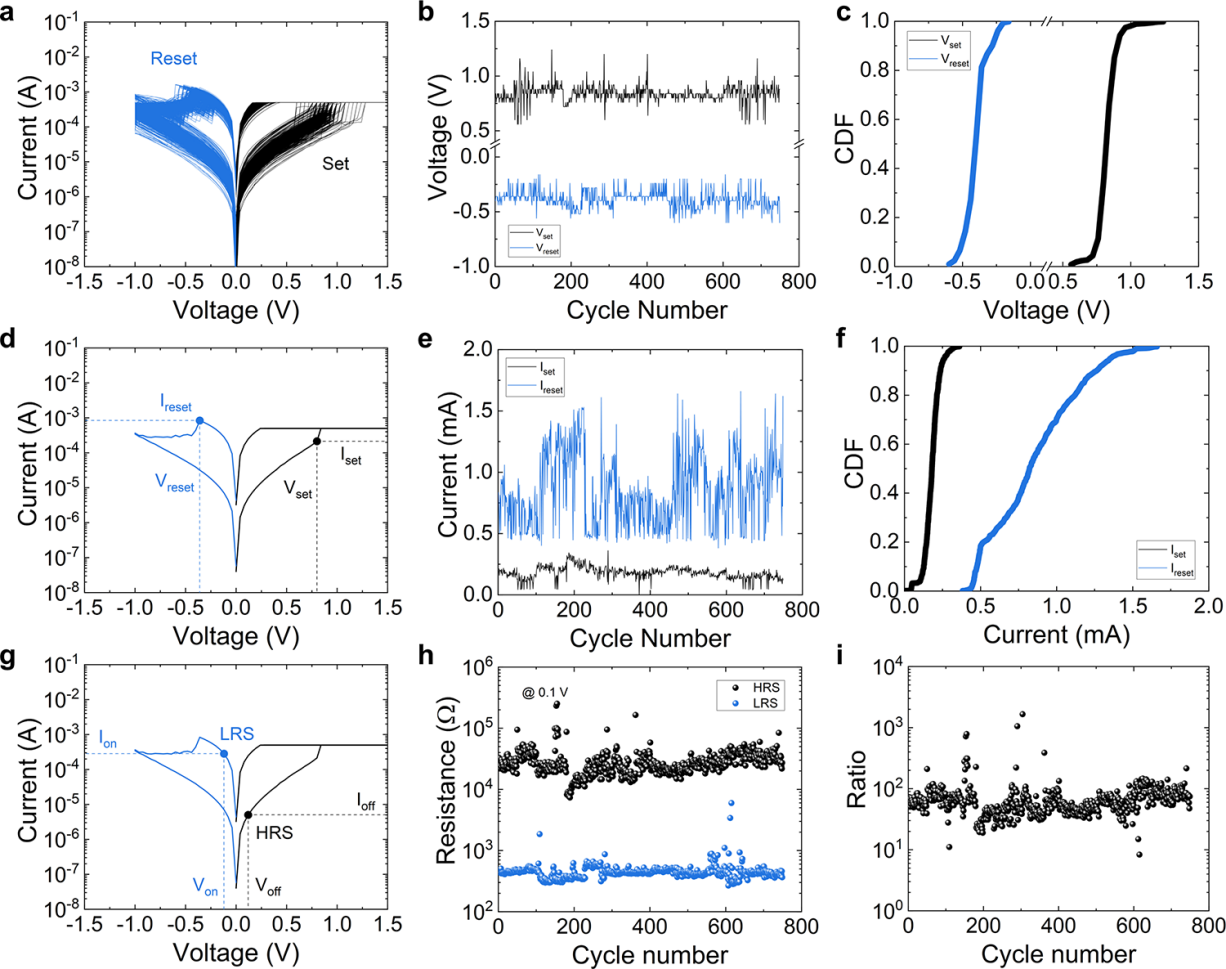
(a) Experimental current versus voltage for the 749 cycles measured applying a ramped voltage stress in the device based on the Au/Ti/TiO_2_/Au stack. (b) Experimental values of *V*_set_ and *V*_reset_ versus cycle number for the long RS series. (c) Cumulative distribution functions for the set and reset voltages. (d) Experimental current versus voltage curve for a single cycle detailing the set and reset voltages and currents points. (e) Experimental values of *I*_set_ and *I*_reset_ versus cycle number for the RS series. (f) Cumulative distribution functions for the set and reset currents. (g) Experimental current versus voltage curve for a single cycle depicting the HRS and LRS points where resistance is obtained. (h) LRS and HRS resistance versus cycle number for all the measured resistive switching series. The data are read at 0.1 V. (i) HRS/LRS resistance ratio versus cycle number calculated from the data corresponding to (h).

The second type of device consisted of Au/Ti/TiO_2_/Au stacks with a lateral size of 5 μm × 5 μm, fabricated on 300 nm SiO_2_/Si wafers. We have adapted the electrode deposition process to the previous fabrication stages. The deposition of the electrodes was done via photolithography, electron beam evaporation, and lift-off, and the deposition of the TiO_2_ film was done by atomic layer deposition. Again, the *I*–*V* curves (749 cycles were measured) are shown in Figure 2a; see that in this case we also have bipolar operation, as in the previous devices. The set and reset voltages and corresponding currents have been extracted in the same way reported above. These parameters have been plotted versus the cycle number in Figures 2b and 2e. The coefficients of variation were also calculated (assuming absolute values) (see Table 1). The CDFs are shown in Figures 2c and 2f; see that a much stronger variability is seen in the reset current and voltage in comparison with the TiO_2_ devices made by anodic oxidation (Figure 1).

The ratio of the HRS/LRS resistances has also been obtained in this case (Figure 2, panels g–i). The results are worse than in the previous case in terms of variability and the *R*_HRS_/*R*_LRS_ ratio obtained, although memory applications would also be possible.

## 3 Results and Discussion

**Figure 3:**
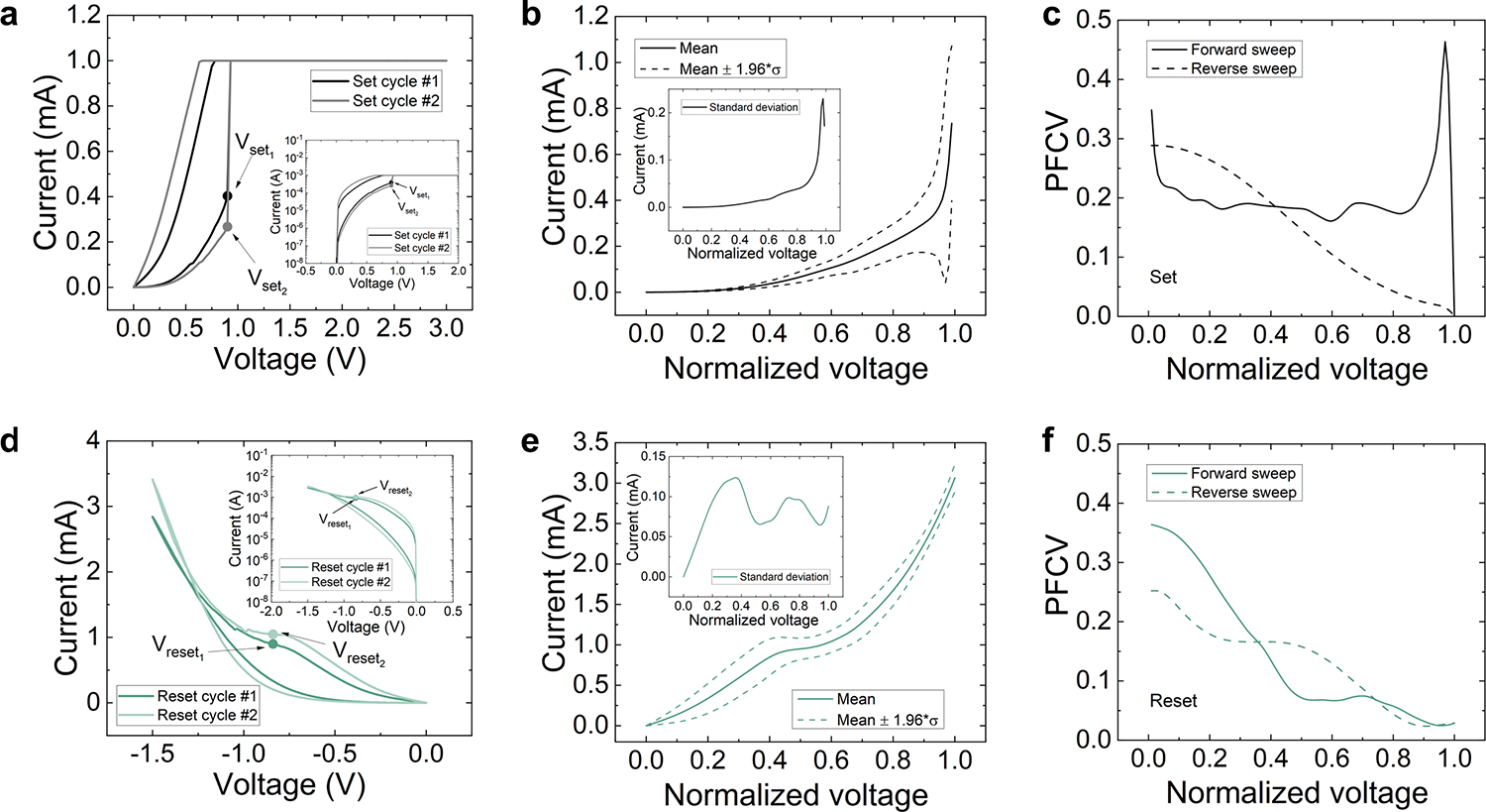
(a) Experimental current versus voltage for two different set curves showing the same set voltage (Pt/TiO_2_/Ti devices). Inset: logarithmic plot for a better visualization. (b) Mean (solid line) and interval (dashed lines) of the normalized curves for the set process. Inset: standard deviation versus normalized voltage. (c) PFCV versus normalized voltage for the set processes (the calculation corresponding to the *I*–*V* section generated by the forward ramps, the proper set process, is shown as a solid line; the calculation corresponding to the *I*–*V* section linked to the reverse ramps, once the set process is performed, is shown as a dashed line). (d) Experimental current versus voltage for two different reset curves showing the same reset voltage. Inset: logarithmic plot for a better visualization. (e) Mean (solid line) and interval (dashed lines) of the normalized curves for the reset process. Inset: standard deviation versus normalized voltage. (f) PFCV versus normalized voltage for the reset processes (the calculation corresponding to the *I*–*V* section generated by the forward ramps, the proper reset process, is shown as a solid line; the calculation corresponding to the *I*–*V* section linked to the reverse ramps, once the reset process is performed, is shown as a dashed line).

In Figures 3a and 3d we show two different *I*–*V* curves (collected in the Pt/TiO_2_/Ti devices) from which the set and reset voltages have been extracted. The values of *V*_set_ and *V*_reset_ are similar, although the shape of the *I*–*V* curves is different from each other (see the logarithmic version of the plots in the corresponding insets). A traditional variability analysis of *V*_set_ and *V*_reset_ (as those reported in refs (28 and 43)) would indicate that the cycle-to-cycle variability is zero. However, using our advanced holistic approach, we can quantify the variability accounting for all the data in the *I*–*V* curves.

To do so, first we normalize the voltage of the *I*–*V* curve to transform all variables in the *X*-axis of the *I*–*V* curves collection (i.e., the voltage) in the [0,1] interval; see Figure S1 and the corresponding explanation of this process in the Supporting Information. The voltage is normalized as follows: |*v*_*ik*_/*v*_*im*_*i*__| (for the *i*th *I*–*V* curve, we have *m*_*i*_ different points in the measured curve) with *k* = 1, 2, ..., *m*_*i*_, where *v*_*im*_*i*__ is the set voltage for the set curves and the final voltage (at the end of the voltage ramp) in the reset processes. The normalization is essential for the functional data analysis process. Nevertheless, the choice of *V*_set_ (the voltage where the current curve jumps up until the compliance value selected in this technology is achieved) and the end of the voltage ramp for the reset process ensure that the complete resistive switching processes are included in the new variability calculation. After normalization, the curves are fitted with B-splines (eqs 1, 2, and 3 in the Supporting Information). Then the *I*–*V* curves are rebuilt with a cubic B-spline basis with 24 functions for each curve in the RS series (Figure S2 shows that this is an optimum choice for a correct accuracy). In this manner, the current can be evaluated at the same points in the normalized voltage interval for all the curves. This procedure was performed, and the current curves for the set and reset processes are plotted in Figures S3a and S3b (B-spline fitted and normalized). This new representation allowed us to calculate the mean *I*–*V* curve, eq 4 (see Figures 3b and 3e), and the standard deviation, eq 5 (see also the insets in Figures 3b and 3e), of the complete data set of curves. This representation also permitted us to plot the current interval were most of the curves are found, i.e., the mean curve ±1.96σ (see the interval in between the dashed lines).

The set and reset correlation plots are shown in Figures S4a and S4b (eq 7). They show in general low autocorrelation among the values of the curves at different normalized voltages. See that for the set processes the higher autocorrelation point is close to a normalized voltage of 1, which means close to the set voltage values. In this respect, the set current at these points shows the higher correlation. However, for the reset current the higher autocorrelation is seen at normalized voltages close to 0.4.

Finally, the pointwise functional coefficient of variation (PFCV) is shown in Figures 3c and 3f for the set and reset processes, respectively. It has been calculated as shown by eq 8. The *I*–*V* curves, both for the set and reset processes, are measured with a first ramp (corresponding to the proper set and reset events, the forward ramp) and a return (reverse) ramp until the voltage signal reaches *V* = 0 V again. Therefore, we need to calculate two PFCV curves: for the *I*–*V* curve sections corresponding to the forward (solid line) and reverse (dashed line) ramps. Because the statistical theory behind the calculations is the functional data analysis, no function with two different current values for a single voltage could be processed; we need to do it separating the results for the forward and reverse ramps. The voltage ranges where the different PFCV curves (solid and dashed lines) dominate are intertwined because the charge transport regimes and the physical mechanisms behind RS change with the applied voltage. The PFCV for the forward ramp (solid line) shows higher values at low normalized voltages for the reset processes (Figure 3f); however, for the second half of the normalized voltage, the PFCV is below 0.1 (the dashed line, for the reverse ramp, behaves in a similar manner); consequently, in this interval the variability is low. The PFCV for the forward ramp for the set processes shows a different behavior (Figure 3c), with higher values at normalized voltages close to 1. See that the conventional CV for the set currents, corresponding to the set voltages, would be obtained at a normalized voltage equals to one. Once the set event occurs, the *I*–*V* curve section for the return ramp (dashed line) shows lower PFCV values for the second half of the normalized voltage interval; this result is reasonable because variability in the LRS (once the set process is over) is lower.

The two-dimensional variability coefficient (2DVC) (which is simply a single-point functional coefficient of variation), the new metric that accounts for the whole set of *I*–*V* curves (including all the data in the curves) by means of a single parameter, has been calculated using eq 9. Because we have two *I*–*V* curve sections, corresponding to the forward and reverse ramps, we can calculate two different single-point functional coefficients of variation (2DVC_f_, for the forward ramp, and 2DVC_r_, for the reverse ramp). A final coefficient (total coefficient) accounting for the contribution of the *I*–*V* curve sections for both types of ramps is 2DVC_t_. See in the Supporting Information the manner 2DVC_t_ is calculated, which is not a simple mean because complex statistical concepts are involved. The values for the 2DVC_t_(set) and 2DVC_t_(reset) are given in Table 1, for both the set and reset processes. With respect to the Pt/TiO_2_/Ti stack, by comparing 2DVC_t_(set) and CV(set), it is seen that variability is low in both cases. However, 2DVC_f_(set) and CV(set) are different; one can find more variability for the set curves (forward ramp, where the set events take place) if we account just for the whole forward *I*–*V* curve data set. The reduction of 2DVC_t_(set) comes from the contribution of 2DVC_r_(set) that corresponds to *I*–*V* curves with higher current values in general (the devices operate in the LRS under the reverse ramp input signal after the set process). Therefore, depending on the ramp considered, the variability changes when all the *I*–*V* curves data are considered; we cannot discriminate this behavior using the conventional 1D CV coefficient. For the reset values (Pt/TiO_2_/Ti stack) the behavior resembles that obtained for the set case. 2DVC_t_ (also 2DVC_f_ and 2DVC_r_) is slightly lower than CV(reset); i.e., a low variability is found through the 2DVC coefficient.

**Figure 4:**
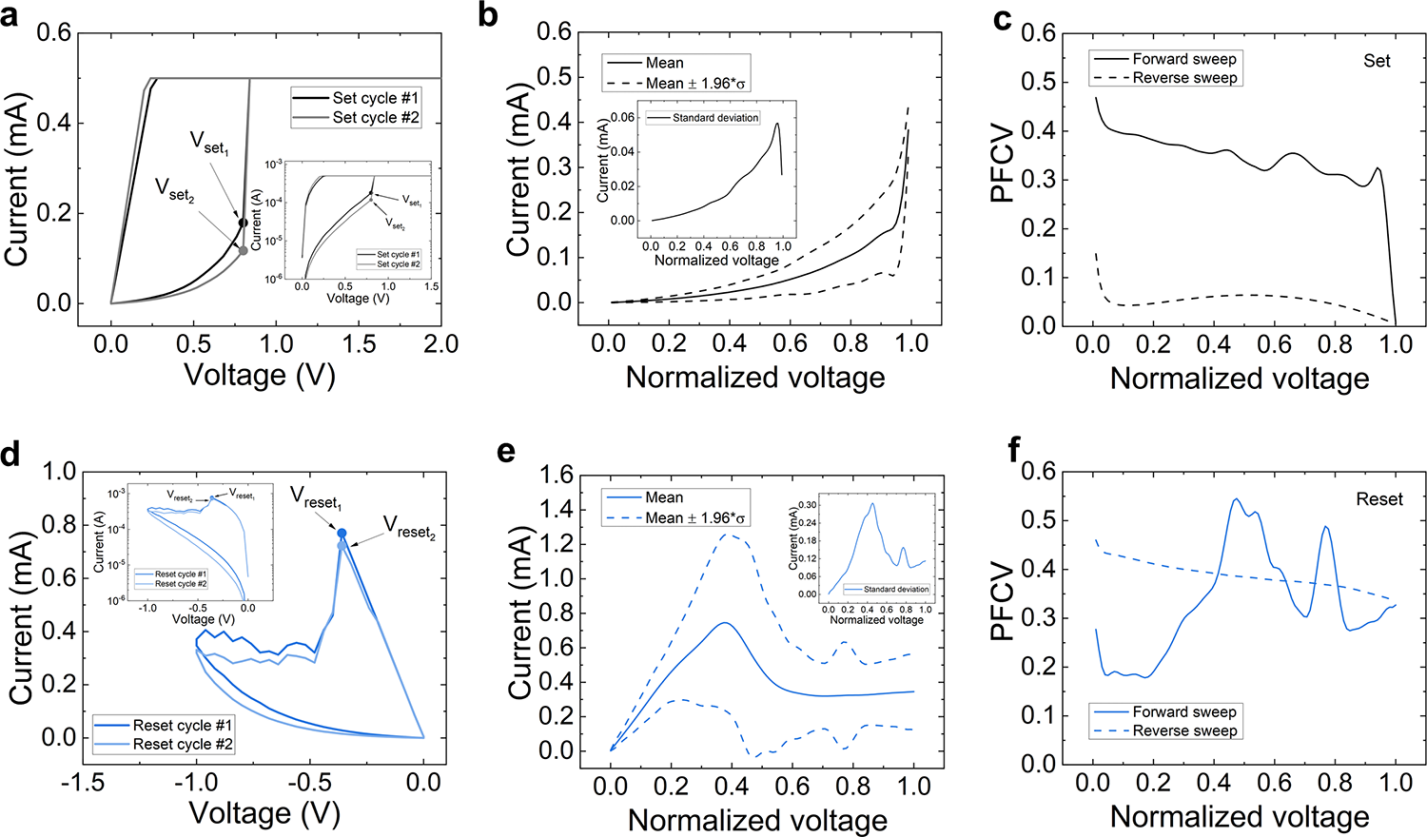
(a) Experimental current versus voltage for two different set curves showing the same set voltage. Inset: logarithmic plot for a better visualization (Au/Ti/TiO_2_/Au devices). (b) Mean (solid line) and interval (dashed lines) of the normalized curves for the set process. Inset: standard deviation versus normalized voltage. (c) PFCV versus normalized voltage for the set processes (the calculation corresponding to the *I*–*V* section generated by the forward ramps, the proper set process, is shown as a solid line; the calculation corresponding to the *I*–*V* section linked to the reverse ramps, once the set process is performed, is shown as a dashed line). (d) Experimental current versus voltage for two different reset curves showing the same reset voltage. Inset: logarithmic plot for a better visualization. (e) Mean (solid line) and interval (dashed lines) of the normalized curves for the reset process. Inset: standard deviation versus normalized voltage. (f) PFCV versus normalized voltage for the reset processes (the calculation corresponding to the *I*–*V* section generated by the forward ramps, the proper reset process, is shown as a solid line; the calculation corresponding to the *I*–*V* section linked to the reverse ramps, once the reset process is performed, is shown as a dashed line).

For the devices based on the Au/Ti/TiO_2_/Au stack, the variability results are given in Figure 4. The study is justified because different *I*–*V* curves could produce the same set and reset voltages (Figures 4a and 4d). The fitted and normalized curves are given in Figures S3c and S3d, and the current mean and standard deviation can be calculated (see Figures 4b and 4e). The autocorrelation for the set curves is also centered at normalized voltages close to 1 and for the reset curves in the 0.4–0.5 interval. In addition, the PFCV is obtained. A high variability is found for the set process in the forward ramp at low normalized values; this high variability is maintained for almost all the cycle, until a normalized voltage close to 0.9 is achieved. The reverse ramps lead to a lower variability in the set curves (Figure 4c). These results are easily seen in Figure 2a, at the sight of the *I*–*V* curves shape. The reset PFCV is higher than in the previous technology considered. In general, a higher variability is seen in the reset curves for this type of device with respect to the previous technology (this can also be confirmed by means of Figure 2a).

We also analyzed the variability by means of the 2DVC metric (Table 1). It can be seen that 2DVC_f_(set) ≫ CV(set); this means that when considering the two-dimensional data set, a much higher variability is obtained with respect to the conventional CV(set). In other words, while it is true that *V*_set_ values can be more grouped (for this reason CV(set) is lower), the set curve section linked to the forward ramps are not uniform with each other (they have different forms). As in the previous case, if the *I*–*V* curves section linked to the reverse ramp are considered (because the current values are much higher for being in the LRS), the 2DVC_t_(set) decreases. 2DVC_t_(reset) and CV(reset) are close; the lower 2DVC_f_(reset) corresponding to curve sections with higher current values compensate for 2DVC_r_(reset) which informs of a higher variability in the curve section linked to the reverse ramp, in comparison to the one-dimensional reset voltage data set.

This new methodology is more rigorous. It clearly reflects numerically the variability we observed in the measured *I*–*V* curves (Figures 1a and 2a); we can distinguish between the different ramps of the voltage signal that lead to groups of curves with a remarkable different variability. The 1D approach does not displays these discrepancies. In the set curves, where the set event is included (forward ramp), variability is much higher than in the 1D case (the 1D case is described by CV(set)), for both technologies. In the reset curves for the Au/Ti/TiO_2_/Au devices the discrepancies between forward and reverse ramps are also seen.

The 2DVC_f_(set) for the Pt/TiO_2_/Ti stack is lower than for the Au/Ti/TiO_2_/Au stack. In addition, 2DVC_t_(reset) (which accounts for both 2DVC_f_ and 2DVC_r_) is much higher in the case of the Au/Ti/TiO_2_/Au stack. This means that in this latter technology, in general, the density of defects is lower. As shown by means of kinetic Monte Carlo simulations,^34,44^ when the compactness of the percolation paths that contribute to resistive switching is lower, a change in the defect density (a low number of oxygen vacancies are recombined; in addition, metal cations can also be involved) leads to a higher variation in the device resistance and therefore to a higher variability. If dense percolation paths are formed, when they are broken at their weakest point, the filament remnants remain stable and lead to lower variability because the HRS current is channelized through the broken filament and, also, the formation of new ones is facilitated in the gap between the filament tip and the electrode, reducing variability. This effect is also produced when an accumulation of defects at one of the electrode interfaces modulates an energy barrier that controls the main charge conduction mechanism (e.g., Schottky emission, Fowler–Nordheim tunneling, etc.); this would also diminish the device variability in an area-dependent charge conduction. In filamentary resistive switching, the defects allow charge transport by hopping and/or trap-assisted tunneling;^45^ if the defect density is very high, charge conduction could be considered ohmic-like.^45,46^ See that both 2DVC_f_ and 2DVC_r_ are higher for Au/Ti/TiO_2_/Au in the reset process. Nevertheless, as can be seen in Figures 1i and 2i, both technologies are appropriate for nonvolatile memory applications.

Finally, the Stanford compact model (SM) was employed to fit the experimental curves corresponding to the Au/Ti/TiO_2_/Au devices. A modified implementation of the variability model in the SM is proposed to reproduce the experimental variability characterized by this new technique and described in Figure 4. The mathematical details of the new implementation are given in the Supplementary Note 2. The new variability obtained with the model reproduces well the experimental data (see Figure S5c); on the contrary, the conventional variability model included in the SM does not perform well when compared to the experimental data (see Figure S5b).

## 4 Conclusions

A variability study is extremely useful to assess the RRAM industrial potential; in addition, it is essential for a correct modeling of variability^31,35,36,47−52^ and thermal effects.^34,53−58^ In this respect, we introduce a novel procedure to evaluate the variability of measured data on RRAMs. Unlike the classical approaches that only use a single point for each *I*–*V* curve that corresponds to *V*_set_ or *V*_reset_ (which would produce the loss of important information such as the trend and shape of the curves), the new methodology includes the complete behavior of set and reset *I*–*V* curves during the analysis; i.e., it is a holistic approach. The variability analysis presented here can be employed to improve electronic design automation (EDA) tools for circuit simulation. The results can be viewed in the context of previous advanced mathematical tools that are needed to correctly analyze RRAM experimental data.^36,59−66^

## Supporting Information Available

Functional coefficient of variation calculation; compact modeling, a new variability implementation (PDF)
