# Supplementary material for: Holistic Variability Analysis in Resistive Switching Memories Using a Two-Dimensional Variability Coefficient
Source: ACS Appl Mater Interfaces. 2023 Apr 7;15(15):19102–10. doi: 10.1021/acsami.2c22617 (PMC10119851; doi:10.1021/acsami.2c22617)
Supplement: Supplementary file 1 — am2c22617_si_005.pdf [file am2c22617_si_005.pdf]

Supplementary information

Holistic variability analysis in resistive switching memories using a two-dimensional variability coefficient

Christian Acal[1], David Maldonado[2], Ana M. Aguilera[1], Kaichen Zhu[3,4], Mario Lanza[3,*], Juan Bautista Roldán[2]

[1]Departamento de Estadística e Investigación Operativa e Instituto de Matemáticas (IMAG), Universidad de Granada, Facultad de Ciencias, Avd. Fuentenueva s/n, 18071 Granada, Spain

[2]Departamento de Electrónica y Tecnología de Computadores. Universidad de Granada. Facultad de Ciencias. Avd. Fuentenueva s/n, 18071 Granada, Spain.

[3]Physical Science and Engineering Division, King Abdullah University of Science and Technology (KAUST), Thuwal 23955-6900, Saudi Arabia.

[4]Department of Electronic and Biomedical Engineering, Universitat de Barcelona, Martí i Franquès 1, E-08028 Barcelona, Spain

* Corresponding authors' email: jroldan@ugr.es, mario.lanza@kaust.edu.sa

Supplementary Note 1: Functional coefficient of variation calculation

We describe here the methodology for this new functional variability analysis in a formal manner to allow the reader to understand the steps followed in the calculations shown in the main manuscript.

Let $I_1(v), \dots, I_n(v)$ be a set of curves belonging to the Hilbert space $L^2[V]$ of squared integrable functions with $\langle f|g\rangle = \int_V f(v)g(v)dv, \ \forall f, g \in L^2[V]$. This set of curves represents the experimental I-V curves measured in a resistive switching series under ramped voltage stress (RVS) operation regime. From a mathematical viewpoint, these curves can be seen as observations of a second order stochastic process $\{I(v): v \in V\}$ and continuous in quadratic mean. This latter requirement implies, $\lim_{h\to 0} E\left[\left(I(v+h) - I(v)\right)^2\right] = 0, \ \forall v \in V$.

The property of continuity in quadratic mean guarantees the continuity of the covariance function [Todorovic1992], which is crucial in many of the functional techniques, e.g., to obtain the spectral decomposition of the covariance operator required in Functional Principal Component Analysis (FPCA). In the context of resistive random access memories, FPCA based on Karhunen-Loève expansion was employed to describe the stochastic evolution of the main features related to reset curves in [AguileraMorillo2019, Aguilera2021, Ruiz-Castro2021].

The two main issues found in practice for the proposed variability analysis are the following:

1. The curves are not defined in the same domain because each curve has a different set/reset point or a different return point in the ramped voltage signal employed. This aspect is very important in the context of functional data analysis; therefore, "*normalization*" of the curve domain is needed. Since we are dealing with I-V curves measured under RVS, we have to use a normalized voltage.

2. The curves are observed in a discrete approach (non continuous), taking into consideration a finite grid of voltage values $\{v_{i1}, v_{i2}, \dots, v_{im_i}\}$ with v_{im_i} being the voltage point where the ith curve is cut. Notice that the voltage values are ranked in increasing order.

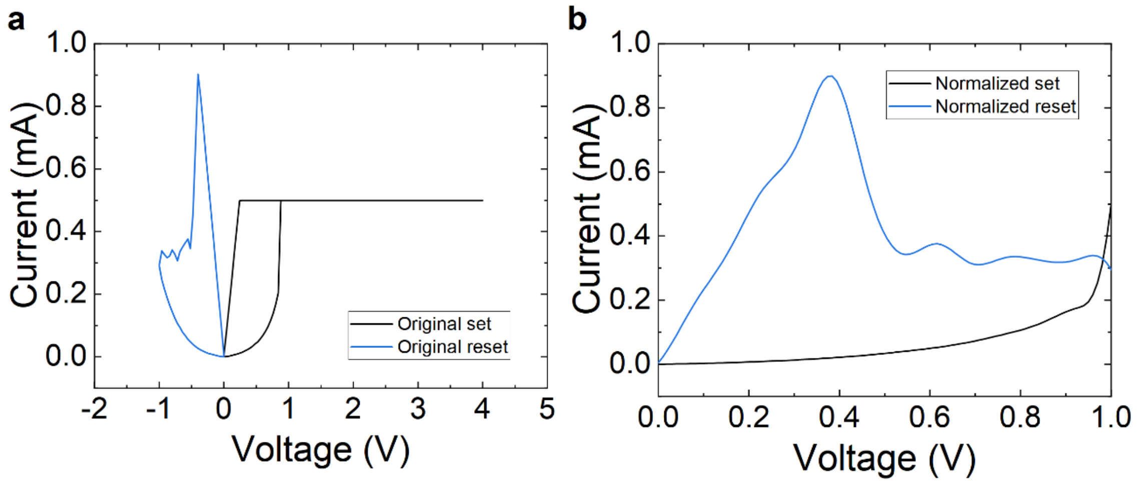

Figure S1. (a) Original experimental I-V curves for a set and reset process, (b) normalized I-V curves, as needed for the new variability study.

For the I-V curve domain normalization process (on the abscissa axis), the registration in the interval [0,1] is achieved by just dividing $v^*_{ik} = v_{ik}/v_{im_i}$ with $k = 1, 2, \dots, m_i$ (see, e.g., [Aguilera2021]). Hereinafter, the I-V curves defined in the interval [0,1] (see Figure S1) will be denoted as $\{x_i(v^*): i = 1, \dots, n; v^* \in [0,1]\}$. See that n is the number of curves measured in the resistive switching series. For the Pt/TiO_2/Ti devices n equals 3900 and for the Au/Ti/TiO_2/Au devices n equals 749.

Once the curves are normalized, the second step of the analysis is performed. To do so, it is usual to consider a finite number of basis functions $\{\phi_1(v^*), \dots, \phi_p(v^*)\}$ to obtain the real functional form of the sample I-V curves we are employing for the variability study [Ramsay2005, Acal2022]. In this manner, the normalized I-V curves can be expressed as follows,

$$x_i(v^*) = \sum_{j=1}^{p} a_{ij}\phi_j(v^*), \quad i = 1, \dots, n, \tag{1}$$

where a_{ij} are the basis coefficients (random variables with finite variance). Generalized cross validation can be used to determine the dimension p of the basis of functions [Craven1978], whereas the choice of basis type depends on the I-V sample curves features. The most useful basis systems are Fourier functions for periodic data, B-Spline basis for non-periodic and smooth paths and wavelets basis for curves with a strong local behaviour. Given that B-Splines bases are considered in the current manuscript, a brief summary is given next:

Let $\tau_0 < \cdots < \tau_r$ be a partition of knots (points) in the interval [0,1] in the abscissa of the I-V curve. More knots can be added to the partition as $\tau_{-q} < \cdots < \tau_{-2} < \tau_{-1} < \tau_0 < \cdots < \tau_r < \tau_{r+1} < \tau_{r+2} < \cdots < \tau_{r+q}$. Then, a B-spline basis of degree q can be iteratively defined by

$$B_{j,q+1}(v^*) := \frac{v^* - \tau_{j-2}}{\tau_{j+q-2} - \tau_{j-2}} B_{j,q}(v^*) + \frac{\tau_{j+q-1} - v^*}{\tau_{j+q-1} - \tau_{j-1}} B_{j+1,q}(v^*),$$
$$q = 1,2, \dots; j = -1,0,1, \dots, r - q + 4, \tag{2}$$

with

$$B_{j,1}(v^*) := \begin{cases} 1 & \tau_{j-2} \le v^* < \tau_{j-1} \\ 0 & otherwise \end{cases} \quad j = -1,0,1, \dots, r + 4.$$

A deep review about these and other bases, as well as their handling in the statistical software R can be checked in [Ramsay2005, Ramsay2009]. In particular, a cubic B-splines basis ($q = 3$) is a suitable option for the set/reset curves, given that these curves do not usually exhibit too much noise. Then, the B-spline basis coefficients for each curve are estimated by the least squares method as,

$$\hat{\boldsymbol{a}}_i = \left(\boldsymbol{\Phi}_i^t \boldsymbol{\Phi}_i\right)^{-1} \boldsymbol{\Phi}_i^t x_i, \qquad (3)$$

with $\boldsymbol{a}_i = \left(a_{i1}, \dots, a_{ip}\right)^t$, $\boldsymbol{\Phi}_i = \left(\phi_j(v_{ik}^*)\right)_{m_i \times p}$ and $x_i = \left(x_{i1}, x_{i2}, \dots, x_{im_i}\right)^t$, where $x_{ik} = x_i(v_{ik}^*); k = 1,2, \dots, m_i$.

The experimental I-V curves that we have employed in this study, for two different technologies, were reproduced by a cubic B-spline basis with 24 functions for each curve after normalization (Figure S2 shows that this is an optimum choice for a correct accuracy).

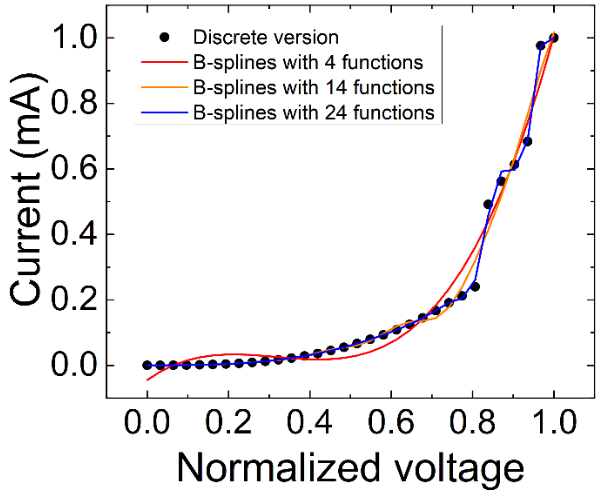

Figure S2. Normalized I-V curve and the fitting obtained with a cubic B-spline basis. As the number of functions in the basis increases, the accuracy of the fit improves significantly.

We performed the rebuilding process for all the curves in the RS series obtained in the laboratory. The results are shown in Figure S3 for the set and reset curves after the normalization process.

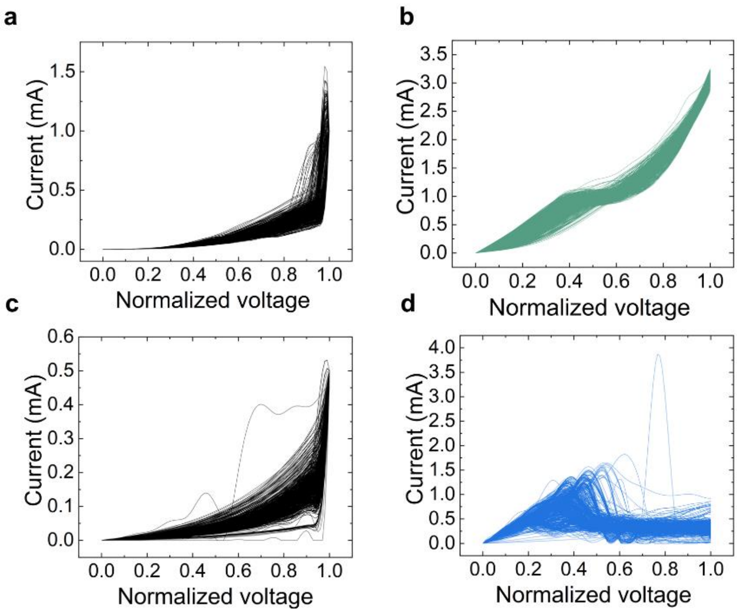

Figure S3. a) Current versus normalized voltage set cycle for the Pt/TiO_2/Ti devices, b) current versus normalized voltage reset cycle for the Pt/TiO_2/Ti devices. c) Current versus normalized voltage set cycle for the Au/Ti/TiO_2/Au devices, d) current versus normalized voltage reset cycle for the Au/Ti/TiO_2/Au devices.

Several classic tools used in exploratory analysis (the classical unidimensional statistical analysis commonly employed in conventional studies) are generalized to the functional framework (more details about descriptive statistics of functional data can be found in [Shang2015]). We have summarized some of them below. See that we change the domain from a one-dimensional dataset (e.g. reset voltages) to a two-dimensional dataset (e.g. the I-V curves for the reset processes).

1.-Sample mean function

$$\bar{x}(v^*) = \frac{1}{n}\sum_{i=1}^{n} x_i(v^*) = \frac{1}{n}\sum_{i=1}^{n}\sum_{j=1}^{p} a_{ij}\phi_j(v^*) = \sum_{j=1}^{p} \bar{a}_j\phi_j(v^*), \quad \forall v^* \in [0,1], \tag{4}$$

with $\bar{a}_j = \frac{1}{n}\sum_{i=1}^{n} a_{ij}$.

2.-Sample variance function

$$\sigma^2(v^*) = \frac{1}{n-1}\sum_{i=1}^{n}\left(x_i(v^*) - \bar{x}(v^*)\right)^2, \quad \forall v^* \in [0,1]. \tag{5}$$

3.- Sample covariance function

$$C(u^*,v^*) = \frac{1}{n-1}\sum_{i=1}^{n}\big(x_i(u^*) - \bar{x}(u^*)\big)\big(x_i(v^*) - \bar{x}(v^*)\big), \;\; \forall u^*, v^* \in [0,1]. \qquad (6)$$

4.- Sample correlation function

$$r(u^*,v^*) = \frac{\hat{C}(u^*,v^*)}{\sqrt{\hat{C}(u^*,u^*)\hat{C}(v^*,v^*)}}, \;\; \forall u^*, v^* \in [0,1]. \qquad (7)$$

The correlation plots corresponding to the r function described in Equation 7 are presented in Figure S4. They show in general low autocorrelation among the values of the curves at different normalized voltages, although the obtained data are nonuniform, see higher autocorrelation values at normalized voltages close to 1 for the set processes.

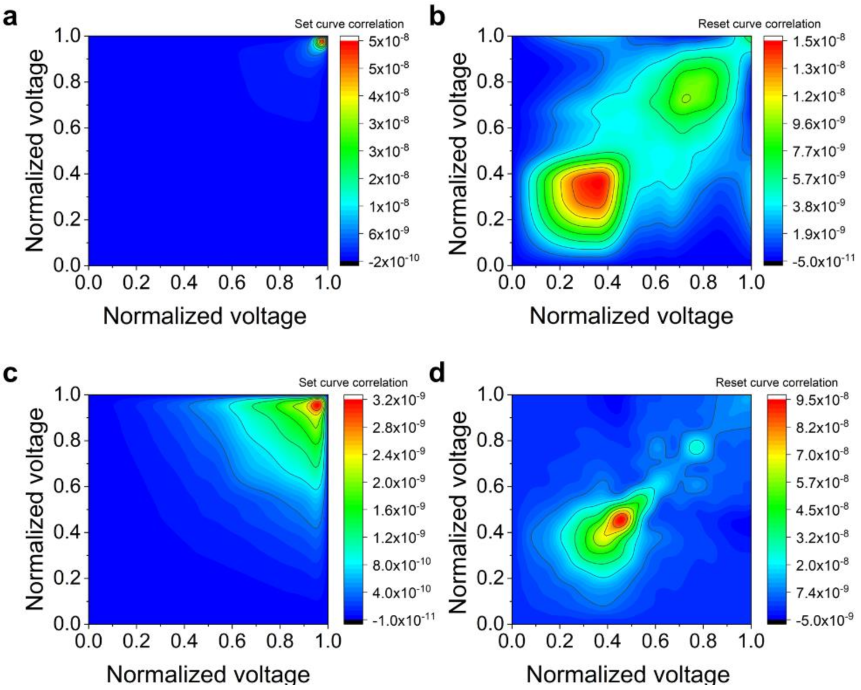

Figure S4. a) Contour plots corresponding to the correlation surfaces for the set processes in Pt/TiO_2/Ti devices, b) Contour plots corresponding to the correlation surfaces for the reset processes Pt/TiO_2/Ti devices. c) Contour plots corresponding to the correlation surfaces for the set processes in the Au/Ti/TiO_2/Au devices, d) Contour plots corresponding to the correlation surfaces for the reset processes in Au/Ti/TiO_2/Au devices.

The functional coefficient of variation (FCV) can be considered to assess the variability of a set of curves, which is the case here. In this work, the classical coefficient of variation ($\mathrm{CV} = \sigma/\mu$) is employed for the one-dimensional dataset and the FCV for the two-dimensional dataset. We are going to analyse here variability accounting for the whole set of I-V curves. This is a much

accurate methodology than the one-dimensional statistical study of the set/reset voltages or any other parameter because of we are taking the complete curves (I-V curves) into account.

Similar to the classical coefficient of variation, the FCV can be used even when the functional variables are expressed in different units because it is dimensionless. As far as we know, for the FCV calculation, two different approaches are available in the literature depending on the final purpose.

a.- [Kocakoç2006] introduced a new FCV to measure how the variability evolves pointwise (pointwise functional coefficient of variation, PFCV), that is, the coefficient of variation is computed for each voltage point v^* in the interval [0,1]. This measure is defined as,

$$\mathrm{PFCV}(v^*) = \frac{\sqrt{\sigma_x^2(v^*)}}{\bar{x}(v^*)}. \tag{8}$$

b.- [Krzysko2019] proposed a multivariate FCV. This new measure provides a single value representing the total variability in the whole domain (a single-point functional coefficient of variation that we name two-dimensional variability coefficient (2DVC) for convenience). By considering the basis expansion of the I-V curves defined in Equation (1), they adapt the multivariate coefficient of variation of Albert-Zhang type (see [Albert2010] for more details) as follows,

$$\mathrm{2DVC} = \sqrt{\frac{\bar{\boldsymbol{a}}^t \boldsymbol{\Psi} \boldsymbol{\Sigma}_{\boldsymbol{a}} \boldsymbol{\Psi} \bar{\boldsymbol{a}}}{(\bar{\boldsymbol{a}}^t \boldsymbol{\Psi} \bar{\boldsymbol{a}})^2}}\,, \tag{9}$$

where $\boldsymbol{\Psi} = \int_0^1 \phi_k(v^*)\phi_j(v^*)\,dt$ is the matrix (order $p \times p$) of inner products between basis functions, $\boldsymbol{\Sigma}_{\boldsymbol{a}}$ and $\bar{\boldsymbol{a}}$ are the covariance matrix (order $p \times p$) and the mean vector (order $p \times 1$) of basis coefficients, respectively. To determine the 2DVC value, the parameters $\boldsymbol{\Sigma}_{\boldsymbol{a}}$ and $\bar{\boldsymbol{a}}$ can be estimated by means of classical and robust estimators [Albert2010, Davies1987, Rousseeuw1985].

The main advantage of robust estimators in comparison with the classic ones is that the formers have a better performance when there are many outlying observations in the sample. The presence of outliers might have an important influence in the modeling of functional data. Multiple approaches have been developed with the goal of identifying functional outliers [Hyndman2007, Febrero2008]. Finally, the R-package *rainbow* contains functions for visualizing functional data and identifying outlier curves from different perspectives [Hyndman2010].

Calculation of the functional coefficient of variation for the bivariate case

In this section we consider the I-V curves measured for the forward ramp and for the reverse ramp. In this respect, we would have a function with two different current values for the same voltage. This cannot be considered in the simple functional data analysis. Therefore, we adapt the theoretical background with the bivariate case (the manner to consider the two I-V curve sections).

The previous methodology is only useful for the situation in which one functional variable is considered (the voltage (corresponding to the X axis) sweeps between 0V to V_{set} for the set

process and 0V to V_{ramp} for reset). However, as described above, the experimental I-V curves have different sections corresponding to the forward and reverse voltage ramps, both for the set and reset processes. Hereinafter, it is supposed that the forward voltage ramp denotes a functional variable and the reverse voltage ramp another variable. This situation is known as a functional bivariate case.

Let $\mathbf{x}_1(v^*), \dots, \mathbf{x}_n(v^*)$ be a functional sample from a bivariate random process denoted as $\mathbf{X}(v^*) = \left(X_1(v^*), X_2(v^*)\right)^t, v^* \in [0,1]$. The $X_*(v^*)$ variable would correspond to the device current at voltage v^*. Note that $\mathbf{x}_i(v^*) = \left(x_{i1}(v^*), x_{i2}(v^*)\right)$. We assume that the process $\mathbf{X}(v^*)$ belongs to the Hilbert space $L_2^2[0,1]$ with the usual characteristics defined above. Taking the basis expansion into account, each component of $\mathbf{X}(v^*)$ can be expressed as follows,

$$X_j(v^*) = \sum_{l=1}^{p_j} a_{jl}\phi_{jl}(v^*), \quad j = 1,2, \tag{10}$$

$p_j \in \mathbb{N}$ may be different for each functional variable. In matrix notation, Equation 10 adopts the following expression,

$$\mathbf{X}(v^*) = \mathbf{\Phi}(v^*)\boldsymbol{a}, \tag{11}$$

where $\boldsymbol{a} = \left(a_{11}, \dots, a_{1p_1}, a_{21}, \dots, a_{2p_2}\right)^t$, and $\mathbf{\Phi}(v^*) = \mathrm{diag}\left(\boldsymbol{\phi}_1^t(v^*), \boldsymbol{\phi}_2^t(v^*)\right)$ is the block diagonal matrix of $\boldsymbol{\phi}_j^t(v^*) = (\phi_{j1}, \dots, \phi_{jp_j})$ with $j = 1,2$. In terms of the functional sample, Equation 11 can be rewritten as,

$$\mathbf{x}_i(v^*) = \mathbf{\Phi}(v^*)\boldsymbol{a}_i, i = 1, \dots, n. \tag{12}$$

If the matrix $\mathbf{\Psi}_{\mathbf{\Phi}}^{1/2}$ exists, where $\mathbf{\Psi}_{\mathbf{\Phi}} = \mathrm{diag}\left(\mathbf{\Psi}_{\phi_1}, \mathbf{\Psi}_{\phi_2}\right)$, and $\mathbf{\Psi}_{\phi_j} = \int_0^1 \boldsymbol{\phi}_j(v^*)\boldsymbol{\phi}_j^t(v^*)\,dt$ being the cross product matrix with order $p_j \times p_j$ corresponding to the basis $\left\{\phi_{jl}\right\}_{l=1}^{p_j}, j = 1,2$; the coefficient 2DVC shown in Equation 9 can be extended to the bivariate case (i.e., accounting for the I-V curve sections corresponding for the forward and reverse voltage ramps at once) as follows (see [Krzysko2019]),

$$2\mathrm{DVC} = \sqrt{\frac{\overline{\boldsymbol{a}}^t\mathbf{\Psi}_{\mathbf{\Phi}}\mathbf{\Sigma}_a\mathbf{\Psi}_{\mathbf{\Phi}}\overline{\boldsymbol{a}}}{\left(\overline{\boldsymbol{a}}^t\mathbf{\Psi}_{\mathbf{\Phi}}\overline{\boldsymbol{a}}\right)^2}}\ . \tag{13}$$

Making use of the previous developments, we could calculate the $2DVC_f$ and $2DVC_r$ coefficients described in the manuscript by means of Equation 9, and $2DVC_t$ coefficient by means of Equation 13.

Supplementary Note 2: Compact modelling, a new variability implementation

We have used the Stanford Model (SM) [Guan2012, Chen2015, Jiang2016] to describe variability, accounting for the results of the methodology developed in the supplementary note 1. This model assumes filamentary conduction, and it calculates the evolution of a conductive nanofilament (CNF) in the dielectric; in particular, the CNF gap, g, i.e. the distance between the CNF tip and the electrode. In this manner, the device resistance evolution is calculated (the implementation is given in Verilog-A [Stanford model]) and the device operation can be described both in the quasi-static and transient operation regimes [Guan2012, Chen2015a, Jiang2016]. In the Chua's modeling formalism, the state variable is the CNF gap [Chua1971]. The abrupt shape of the Au/Ti/TiO_2/Au device I-V curves suggests filamentary conduction; other TiO_2-based devices have also been found to show filamentary conduction which is mainly due to the formation and rupture of nanometric paths (conductive nanofilaments) with a high concentration of oxygen vacancies that show ohmic-like charge conduction features [Lee2017, Carta2016].

The dynamics of the state variable permits set and reset processes, and allows the switching between the LRS and HRS. The conductive filament temperature is calculated with a simplified version of the heat equation. The model simplicity and versatility make it the choice of many research groups to include resistive memories in their circuit designs. There are other models available, although some of them are modifications of the SM [Gonzalez-Cordero2016, Huang2013, Huang2017, Jimenez-Molinos2015, Bocquet2014].

The SM can be employed to reproduce experimental I-V curves after parameter fitting [Guan2012, Chen2015a, Jiang2016, Maldonado2021]. For the variability section of the model, there are specific parameters: δ_g^0, T_{crit}, T_{smth}. They are used to implement a correction to g, the CNF gap, see Equation 14,

$$\delta_g(T) = \frac{\delta_g^0}{\left\{1 + exp\left[\frac{T_{crit} - T}{T_{smth}}\right]\right\}} \tag{14}$$

where T stands for the CNF temperature [Guan2012]. The CNF gap variation (the model state variable) is calculated as follows,

$$g_{t+\Delta t} = \int \left(\frac{dg}{dt} + \delta_g(T) \times \chi(t)\right) dt \tag{15}$$

where $\delta_g(T)$ accounts for random variations, $\chi(t)\ is$ a Gaussian distributed noise with zero-mean and root mean square of unity [Jiang2016] generated at each time step. The dynamics of the CNF gap is given in Equation 16.

$$\frac{dg}{dt} = -\, v_0 e^{\frac{-E_{g,m}}{k_B T}} \sinh\left(\frac{\gamma(g) a_0 q V_{RRAM}}{t_{ox} k_B T}\right) \tag{16}$$

in which t_{ox} stands for the dielectric thickness, E_g is the activation energy for vacancy generation in the set process, E_m is the energy barrier for oxygen ion migration that controls the reset process, v_0 is the velocity dependent on the attempt-to-escape frequency, a_0 the hopping site

distance and V_{RRAM} the applied voltage, which drops mainly at the CNF gap, γ is the field local enhancement factor that accounts for the polarizability of the material [Jiang2016].

In Figure S5a we plot the experimental curves from Figure 2a and the mean curve as obtained from Equation 4. Notice that after calculating the mean curve we have de-normalized it to plot it along the original experimental curves. A correct mean curve cannot be obtained without the previous normalization process, since we would sum currents limited by the compliance current (after the set event) with current values corresponding to voltages below the set voltages. The SM has been tuned to fit the mean curves obtained in Figure 4b and 4e. The parameter set employed is given in Table 1. The values are in line with previous works [Guan2012, Chen2015a, Jiang2016, Maldonado2021].

Stanford model parameters			
**Device parameters**	**Unit**	**Set**	**Reset**
T_{ox}	nm	10	
V_0	V	0.2	
I_0	mA	15	6
g_0	nm	0.2	
v_0	m/s	5×10^6	
α	-	1	0.6
E_a / E_m	eV	1	1.4
δ_g^0	nm	0.3	0.6
T_{smth}	K	400	
T_0	K	300	
T_{crit}	K	450	
g_{min}	nm	0.5	
g_{max}	nm	1.5	
g_{ini}	nm	1.5	

Table 1. Parameters employed to fit the mean set and reset curves calculated previously.

The conventional SM implementation for variability is described in Equations 14 and 15, where the values of δ_g^0, T_{smth} presented in Table 1 are in line with ref. [Roldan2023]. It can be seen in Figure S5b that the variability is not well modeled since the variation of the curves is low and the set and reset values are not well reproduced. Taking into consideration the limitations of this model for our devices, we modified the SM variability implementation to obtain curves in the interval marked with the dashed lines shown in Figures 4b and 4e in the main manuscript. To do so, in addition to account for Equations 14 and 15, we included variations of the I_0 parameter by generating random numbers in the [0,3] interval (uniform distribution) to redefine I_0 parameter (I_0'), as described in Equation 17,

$$I_0' = I_0 + (I_0 * rnd) \quad (17)$$

Furthermore, we allowed variations above g_{max} for the gap in the set process that lead to higher variability in the HRS. The results obtained with the new implementation are shown in Figure

S5c. As can be seen, the variability corresponds much more accurately to the one characterized by the new statistical technique developed here.

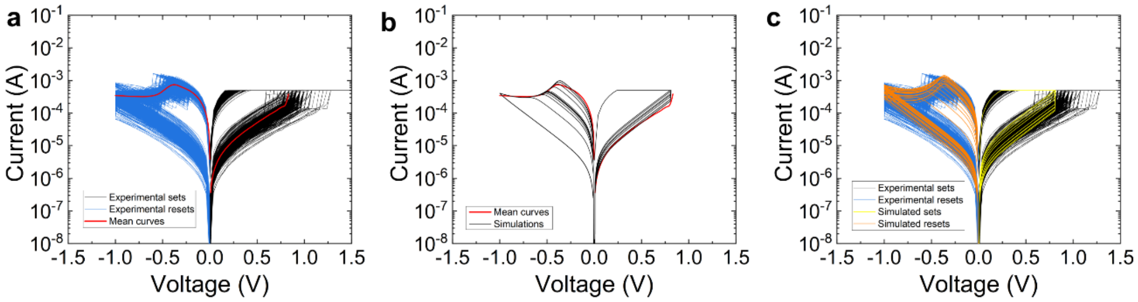

Figure S5. a Experimental I-V curves for the Au/Ti/TiO_2/Au devices and mean (red line) curves (see Figures 4b, 4e in the main manuscript) for the set and reset processes. **b** SM simulated curves along with the mean (red line) curves, including the standard variability implementation in the SM. **c** Experimental and SM simulated I-V curves for the set and reset processes including the new model implemented to allow the variability characterized by the statistical procedure developed in this manuscript.

References

[Acal2022] Acal, C., & Aguilera, A. M., "Basis expansion approaches for functional analysis of variance with repeated measures", *Advances in Data Analysis and Classification*, 1-31, 2022.

[Aguilera2021] Aguilera, A. M., Acal, C., Aguilera-Morillo, M. C., Jiménez-Molinos, F., & Roldán, J. B., "Homogeneity problem for basis expansion of functional data with applications to resistive memories. *Mathematics and Computers in Simulation*, *186*, 41-51, 2021.

[AguileraMorillo2019] Aguilera-Morillo, M. C., Aguilera, A. M., Jiménez-Molinos, F., & Roldán, J. B. (2019). Stochastic modeling of Random Access Memories reset transitions. *Mathematics and Computers in Simulation*, *159*, 197-209.

[Albert2010] Albert, A., & Zhang, L. "A novel definition of the multivariate coefficient of variation", *Biometrical Journal*, *52*(5), 667-675, 2010.

[Bocquet2014] Bocquet M, Deleruyelle D, Aziza H, Muller C, Portal J-M, Cabout T and Jalaguier E, "Robust compact model for bipolar oxide-based resistive switching memories", IEEE Trans. Electron Devices, 61, pp. 674–81, 2014.

[Carta2016] D. Carta, I. Salaoru, A. Khiat, A. Regoutz, C. Mitterbauer, N. M. Harrison, and T. Prodromakis, "Investigation of the Switching Mechanism in TiO2-Based RRAM: A Two-Dimensional EDX Approach", ACS Applied Materials & Interfaces, 8 (30), 19605-19611, 2016.

[Chen2015] P. Chen and S. Yu, "Compact Modeling of RRAM Devices and Its Applications in 1T1R and 1S1R Array Design" IEEE Transactions on Electron Devices, vol. 62, no. 12, pp. 4022-4028, Dec. 2015.

[Chua1971] L. Chua, "Memristor-the missing circuit element", IEEE Transactions on circuit theory, 18(5), 507-519, 1971.

[Davies1987] Davies, P. L., "Asymptotic behavior of S-estimators of multivariate location parameters and dispersion matrices", *The Annals of Statistics, 15,* 1269-1292, 1987.

[Febrero2008] Febrero, M., Galeano, P., & González-Manteiga, W. "Outlier detection in functional data by depth measures, with application to identify abnormal NOx levels", *Environmetrics: The official journal of the International Environmetrics Society*, *19*(4), 331-345, 2008.

[Graven1989] Graven, P., "Smoothing noisy data with spline function: estimating the correct degree of smoothing by the method of Generalized Cross-Validaton" *Number. Math.*, *31*, 377-403, 1989.

[Gonzalez-Cordero2016] G. González-Cordero, J.B. Roldán, F. Jiménez-Molinos, J. Suñé, S. Long y M. Liu, "A new model for bipolar RRAMs based on truncated cone conductive filaments, a Verilog-A approach", Semiconductor Science and Technology, 31, p. 115013, 2016.

[Guan2012] X. Guan, S. Yu, and H.-S. Philip Wong, "A SPICE Compact Model of Metal Oxide Resistive Switching Memory With Variations," Electron Device Letters, IEEE, vol.33, no.10, pp.1405,1407, Oct. 2012.

[Huang2013] Huang, P., Liu, X. Y., Chen, B., Li, H. T., Wang, Y. J., Deng, Y. X., … Kang, J. F., "A Physics-Based Compact Model of Metal-Oxide-Based RRAM DC and AC Operations", IEEE Transactions on Electron Devices, 60(12), 4090–4097, 2013.

[Huang2017] Huang, P., Zhu, D., Chen, S., Zhou, Z., Chen, Z., Gao, B., … Kang, J., "Compact Model of HfOX-Based Electronic Synaptic Devices for Neuromorphic Computing", IEEE Transactions on Electron Devices, 64(2), 614–621, 2017.

[Hyndman2007] Hyndman, R. J., & Ullah, M. S. "Robust forecasting of mortality and fertility rates: A functional data approach", *Computational Statistics & Data Analysis*, *51*(10), 4942-4956, 2007.

[Hyndman2010] Hyndman, R. J., & Shang, H. L. "Rainbow plots, bagplots, and boxplots for functional data", *Journal of Computational and Graphical Statistics*, *19*(1), 29-45, 2010.

[Jiang2016] Z. Jiang, Y. Wu, S. Yu, Member, L. Yang, K. Song, Z. Karim, H.-S. P. Wong, "A Compact Model for Metal–Oxide Resistive Random Access Memory With Experiment Verification", IEEE Transactions on Electron Devices, vol. 63, no. 5, pp. 1884-1892, May 2016.

[Jimenez-Molinos2015] F. Jiménez-Molinos, M.A. Villena, J.B. Roldán y A.M. Roldán, "A SPICE Compact Model for Unipolar RRAM Reset Process Analysis", IEEE Transactions on Electron Devices, 62, pp. 955-962, 2015.

[Kocakoç2016] Kocakoç, İ. D., Keser, İ. K., & Şehirlioğlu, A. K., "A new descriptive statistic for functional data: functional coefficient of variation. *Alphanumeric Journal*, *4*(2), 1-10, 2016.

[Krzysko2019] Krzysko, M., & Smaga, Ł. "A multivariate coefficient of variation for functional data. *Statistics and Its Interface"*, *12*(4), 647-658, 2019.

[Lee2017] K.-J. Lee, Y.-C. Chang, C.-J. Lee, L.-W. Wang, Y.-H. Wang, "1T1R nonvolatile memory with Al/TiO2/Au and sol-gel-processed insulator for barium zirconate nickelate gate in pentacene Thin Film Transistor", materials, 10, 1408, 2017.

[Maldonado2021] D. Maldonado, F. Aguirre, G. González-Cordero, A.M. Roldán, M.B. González, F. Jiménez-Molinos, F. Campabadal, E. Miranda, J.B. Roldán, "Experimental study of the series resistance effect and its impact on the compact modeling of the conduction characteristics of HfO2-based resistive switching memories", Journal of Applied Physics, 130, 054503, 2021.

[Ramsay2005] Ramsay, J.O., & Silverman, B.W., "Functional data analysis", Springer, Berlin, 2005.

[Ramsay2009] J.O. Ramsay, G. Hooker, S. Graves, "Functional Data Analysis with R and MATLAB", Springer-Verlag, 2009.

[Roldan2023] J.B. Roldán, E. Miranda, D. Maldonado, A.N. Mikhaylov, N.V. Agudov, A.A. Dubkov, M. N. Koryazhkina, M.B. González, M.A. Villena, S. Poblador, M. Saludes-Tapia, R. Picos, F. Jiménez-Molinos, S. G. Stavrinides, E. Salvador, F.J. Alonso, F. Campabadal, B. Spagnolo, M. Lanza, L.O. Chua, "Variability in resistive memories", Advanced Intelligent Systems, in press, 2023.

[Rousseeuw1985] Rousseeuw, P. J. "Multivariate estimation with high breakdown point", *Mathematical statistics and applications*, *8*(283-297), 37, 1985.

[Ruiz-Castro2021] Ruiz-Castro, J. E., Acal, C., Aguilera, A. M., Aguilera-Morillo, M. C., & Roldán, J. B. (2021). Linear-Phase-Type probability modelling of functional PCA with applications to resistive memories. *Mathematics and Computers in Simulation*, *186*, 71-79.

[Shang2015] Shang, H. L., “Resampling techniques for estimating the distribution of descriptive statistics of functional data”, *Communications in Statistics-Simulation and Computation*, *44*(3), 614-635, 2015.

[Stanford model] https://nano.stanford.edu/downloads/stanford-rram-model, web checked on January 15, 2023.

[Todorovic1992] Todorovic, P., “An introduction to stochastic processes and their applications”, Springer-Verlag, New York, 1992.
